# Enhanced biomethane production rate and yield from lignocellulosic ensiled forage ley by in situ anaerobic digestion treatment with endogenous cellulolytic enzymes

**DOI:** 10.1186/s13068-017-0814-0

**Published:** 2017-05-16

**Authors:** Jutta Speda, Mikaela A. Johansson, Anna Odnell, Martin Karlsson

**Affiliations:** 1 Molecular Biotechnology, Department of Physics, Chemistry and Biology, Linköping University, 581 83 Linköping, Sweden; 2 Karshult Municipal Waste Water Treatment Plant, 591 86 Motala, Sweden; 3 InZymes Biotech AB, Gjuterigatan 1B, 582 73 Linköping, Sweden

**Keywords:** Biogas, Lignocellulose, Cellulolytic, Cellulase, Enzyme, Hydrolysis, Biochemical methane potential (BMP), Anaerobic digestion, In situ

## Abstract

**Background:**

Enzymatic treatment of lignocellulosic material for increased biogas production has so far focused on pretreatment methods. However, often combinations of enzymes and different physicochemical treatments are necessary to achieve a desired effect. This need for additional energy and chemicals compromises the rationale of using enzymes for low energy treatment to promote biogas production. Therefore, simpler and less energy intensive in situ anaerobic digester treatment with enzymes is desirable. However, investigations in which exogenous enzymes are added to treat the material in situ have shown mixed success, possibly because the enzymes used originated from organisms not evolutionarily adapted to the environment of anaerobic digesters. In this study, to examine the effect of enzymes endogenous to methanogenic microbial communities, cellulolytic enzymes were instead overproduced and collected from a dedicated methanogenic microbial community. By this approach, a solution with very high endogenous microbial cellulolytic activity was produced and tested for the effect on biogas production from lignocellulose by in situ anaerobic digester treatment.

**Results:**

Addition of enzymes, endogenous to the environment of a mixed methanogenic microbial community, to the anaerobic digestion of ensiled forage ley resulted in significantly increased rate and yield of biomethane production. The enzyme solution had an instant effect on more readily available cellulosic material. More importantly, the induced enzyme solution also affected the biogas production rate from less accessible cellulosic material in a second slower phase of lignocellulose digestion. Notably, this effect was maintained throughout the experiment to completely digested lignocellulosic substrate.

**Conclusions:**

The induced enzyme solution collected from a microbial methanogenic community contained enzymes that were apparently active and stable in the environment of anaerobic digestion. The enzymatic activity had a profound effect on the biogas production rate and yield, comparable with the results of many pretreatment methods. Thus, application of such enzymes could enable efficient low energy in situ anaerobic digester treatment for increased biomethane production from lignocellulosic material.

## Background

Enzymes are being utilized in an ever increasing number of biotechnological processes. Although established in many other industrial applications, addition of hydrolytic enzymes to increase the rate and yield of anaerobic digestion in biogas production is still not used on a large scale. However, since much of the organic matter used as substrates in biogas production has low biological availability, this industrial biofuel production process may benefit from employing hydrolytic enzymes. In contrast to other biofuel substrates, biogas production does not necessarily involve the use of mono substrates, which makes selection of appropriate enzymes and processes more difficult. Still, there are substrates or suggested substrates that are fairly homogenous, well investigated and characterized. These include substrates such as excess waste activated sludge, micro algae and lignocellulosic plant material, either derived from agricultural waste or grown purposely as biogenic energy crops. All these bioresources are of interest as substrates for increased biogas production but all are also associated with low biogas product yields due to slow hydrolysis in the first step of the biological methanogenesis process. In the case of lignocellulosic substrates, which are produced at a level of 60 billion tons per year [1], the slow hydrolysis originates from the chemical and structural composition of the biomass. The complex structure, characterized by a strong network of mainly crystalline cellulose, hemicellulose and lignin, makes lignocellulose insoluble and recalcitrant to enzymatic hydrolysis [2]. Thus, to achieve anaerobic digestion as completely as possible, very long retention times in the anaerobic digester are required [3, 4], which make the capital costs of large volume digesters high. Part of the recalcitrance to hydrolysis can be attributed to the crystallinity of cellulose, which can only be efficiently hydrolyzed, in its simplest form, by a combination of endo- and exo-glucanases together with β-glucosidases [5]. However, it has been found that the crystallinity of cellulose does not completely preclude hydrolysis because it can proceed if appropriate enzymes can access the crystalline cellulose [1]. Thus, a common opinion is that for an effective process, pretreatments that eliminate structural barriers to enzymatic hydrolysis are necessary in preparation of lignocellulosic biomass for biogas production. These methods are either physical or chemical, including, but not limited to, e.g., comminution, acid hydrolysis, ammonia fiber expansion, steam explosion, etc. [1, 6]. However, these pretreatment methods require expensive auxiliary equipment or chemicals, are very energy intensive and can produce waste streams, making them economically and environmentally unattractive. In contrast, enzymatic treatment offers the advantages that enzymes are active under mild conditions in aqueous solutions and can be used to target specific structures in the substrate. Therefore, the use of enzymes for enhancing biogas production from various substrates is of general interest [7, 8].

Two lines of enzymatic treatment strategies for lignocellulose can be found in the literature. First, pretreatment of lignocellulosic material by laccases and peroxidases to degrade lignin, and thereby make the cellulose accessible to endogenous cellulolytic enzymes in anaerobic digestion. Second, addition of cellulolytic enzymes and other polysaccharases to degrade cellulose and other polysaccharides directly. In the first of these strategies, lignin degradation, pretreatment utilizes enzymes from various fungi [9–11]. These experiments have resulted in increased yields of biogas [9, 10], but also in no significant increase in yield [11]. The lack of effect on yield can be explained by the release of phenolic compounds that negatively influence the rate of biogas production and correlate with the lignin content of the substrate [11]. However, pretreatment with lignin breaking enzymes necessitates pH adjustment of the substrate and addition of co-factors for optimal enzyme activity, and thus much additional treatment besides the enzyme addition itself. The second strategy of enzyme treatment, using cellulases and other polysaccharases, is the main type of pretreatment used to date, either as a standalone enzyme pretreatment or in combination with alkaline pre-enzyme pretreatment. For the latter, this includes treatment with hydroxides for 12–24 h at 20–50 °C, followed by pH adjustment for enzyme treatment by cellulases and other polysaccharases for 24–72 h [12–15], thus requiring a fair amount of additional treatment besides the enzyme treatment itself. However, interestingly, this combined treatment has been shown to increase the kinetics of biogas production as well as the yield [12–14]. This is in contrast to sole laccase/peroxidase pretreatment of lignin, which only increases the yield in biogas production [9, 10]. This implies that the sole laccase/peroxidase treatment does in fact give access to additional substrate that can be degraded by endogenous enzymes in the biogas process, whereas the combination of alkaline pretreatment followed by polysaccharase treatment provides both access to new material (higher yield) and hydrolyzes some of this material before addition to the biogas process (higher rate). Thus, both approaches perform as expected. Trials with standalone pretreatment with cellulases and other polysaccharases for 6 h–7 days at 37–50 °C [3, 16–18] have shown more mixed results, ranging from no effect at all on rate and yield [3] to increased yield only [16, 17], increased rate only [18] or a combination of both increased yield and rate at selected time points [19].

In the examples above [9–19], the biomass was *pretreated* to different degrees. This would, for full-scale implementation, necessitate varying amounts of added energy (for milling and heating/cooling), chemicals (for alkaline treatment and pH adjustments) and equipment (to hold the biomass during pretreatment) in addition to the enzymatic pretreatment. Therefore, these approaches somewhat undermine the rationale of using enzymes in the first place. Thus, to minimize capital and operational expenditure, it would be desirable to be able to add the enzymes directly to the biogas process. This would further alleviate any potential enzyme hydrolysis limitations due to product inhibition from released sugars in closed pretreatment processes [20] because in the anaerobic digester, the released sugars would be continuously consumed by the microorganisms present. However, in several trials of anaerobic digesters with in situ enzyme treatment, no significant effect on biogas production rate and yield was observed [18, 21–23], although positive effects have been reported in batch experiments [24] and full-scale trials [4]. Nevertheless, it should be noted that in the full-scale trials, the increase in biomethane yield was inferred from the amount of biomethane actually produced, as compared to the calculated biomethane potential of the respective substrate mixes investigated, rather than from full experimental data. Thus, the results from adding polysaccharolytic enzymes directly to the biogas process of lignocellulosic material are contradictory with an inclination toward no or a low positive effect. The reason that no effect is sometimes observed has been attributed to, amongst other factors, the limited activity lifetime of the added enzymes in the anaerobic digestion environment [21, 24]. This was recently reported for enzymes added to the anaerobic digester milieu of a waste water treatment plant sludge digestate [25], for which it was concluded that the limited activity lifetime of the added enzymes was due to proteolytic degradation of the added enzymes by endogenous proteases. In addition, some enzymes had low or no activity at all in the anaerobic digester environment, most notably the evaluated cellulases.

These findings are not necessarily surprising because the environment in an anaerobic digester, and probably in certain biogas substrates, can be expected to be more hostile to added enzymes due to high endogenous microbial and proteolytic activity. Thus, the environment for enzymatic pretreatment of pure substrates with low microbial activity, e.g., cereals for bioethanol production, is very different from the environment of in situ treatment in anaerobic digesters. Therefore, adding enzymes to anaerobic digesters, or certain substrates, to promote hydrolysis is only possible if enzymes are available that are evolutionarily adapted to be efficient and have a sufficiently long lifetime under the conditions prevailing in these environments. In this context, it should be noted that the cellulolytic enzymes assessed for both pretreatment and anaerobic digesters in situ treatment all originate from aerobic fungi that are not naturally present in anoxic environments. The predominant enzyme source, when stated, is *Trichoderma reesei* [16–18, 21, 22, 24], while enzymes originating from *Trichoderma longibrachiatum* [16]*, Penicillium funiculosum* [16]*, Humicola* sp. [18, 22], *Aspergillus niger* [18, 21], and *Acremonium* sp. [23] are less frequently used. Thus, commercially available enzymes do not originate from microorganisms included in microbial communities in anaerobic methanogenic habitats, and therefore cannot be expected to be evolutionarily adapted to the conditions of anaerobic digesters. Hence, it is unlikely that they would have the efficiency and lifetime appropriate for use in situ in an anaerobic digester environment.

Anaerobic degradation of lignocellulosic materials can, thus, be concluded to be a slow process. Nevertheless, there are undoubtedly microorganisms in methanogenic microbial communities able to produce cellulolytic enzymes [26]. Therefore, it would make sense to search for these enzymes under the same conditions as those encountered in anaerobic digesters. In contrast to aerobic organisms, anaerobes often degrade cellulose with multienzyme complexes known as cellulosomes. However, some anaerobic microorganisms, such as *Clostridium thermocellum,* are known to produce both released extracellular cellulases and cellulosomes anchored to the cell surface [26]. Therefore, the reason that cellulose degradation is slow in anaerobic digestion may not necessarily be because the enzymes are not sufficiently efficient but because there is not a sufficient amount of enzymes present. Microbial extracellular enzyme production is essentially regulated by the interplay between the cost of releasing nutrients like carbon, nitrogen, and sulfur in produced and secreted enzymes against the benefits of increasing the amount of available nutrients. According to this “evolutionary-economic principle of microbial metabolism” [27], enzyme production should increase when simple nutrients are scarce and complex nutrients are abundant. Thus, for a high enzyme production and cellulose hydrolysis rate, it is not enough that a complex substrate (cellulose) is present, but simple nutrients (sugars) need to be scarce. Notably, the cellulolytic activity has been found to be three to fivefold higher in the feces supernatant of herbivores than in anaerobic digester liquid [28]. This is logical since in herbivores, the fatty acids produced from the fermentation of sugars are continuously withdrawn to the blood stream of the animal, thus driving the consumption of sugars. In contrast, in the closed system of anaerobic digesters, the produced fatty acids need to be consumed solely by slow-growing anaerobic acetogens and methanogens. Thus, although not desirable for efficient biogas production, from the point of view of the microorganisms habituating biogas reactors, cellulose degradation might not need to be faster than it is and no overproduction of microbial cellulolytic enzymes may occur.

In an earlier work performed by us, these limitations imposed by the evolutionary-economic principle of microbial metabolism were used to control the production of extracellular enzymes in a methanogenic microbial community [29]. Briefly, the microbial community in an experimental biogas reactor was continuously fed with a chemically defined medium in which all nutrients were supplied as simple nutrients (glucose, amino acids, fatty acids, etc.) until a metabolic steady-state was reached. Under these conditions, a high cell density was reached and the extracellular enzyme expression was strongly suppressed. From this enzyme suppressed metabolic steady-state, it was possible to induce the desired enzyme activity without crosstalk between the enzymes studied (cellulases and proteases). Thus, the methanogenic community, obviously able to metabolize glucose, responded to the developing deficiency in six carbon sugars and the presence of cellulose by producing cellulolytic enzymes necessary to hydrolyze cellulose under the conditions of anaerobic digestion. It was further found that this high cellulolytic activity led to rapid digestion and a high rate of biogas production from the filter paper added to induce cellulolytic activity once the production of cellulolytic enzymes had commenced. That is, these cellulases, produced by the methanogenic community, were active against cellulose in the anaerobic digester environment and should reasonably be evolutionarily adapted to the conditions prevailing in that environment. Hence, it was hypothesized that these extracellular cellulolytic enzymes may also be active and stable enough to be used for in situ treatment for increased biogas production from lignocellulosic substrates. In the current work, these enzymes were induced, collected, and tested for their effect on biogas production rate and yield on a lignocellulosic substrate in biochemical methane potential (BMP) tests.

## Methods

### Origin of microbial community and cellulolytic enzymes

The methanogenic microbial community, maintained on a chemically defined medium in a constructed environment and used as a source of cellulolytic enzymes, has earlier been described in detail [29]. Briefly, the microbial community originated from a full-scale anaerobic digester treating mixed sources of waste, mainly slaughter house waste, including rumen content. Thus, the original microbial community comprised microorganisms competent in degrading fats, proteins and cellulose.

### Induction of endogenous cellulases

**Fig. 1:**
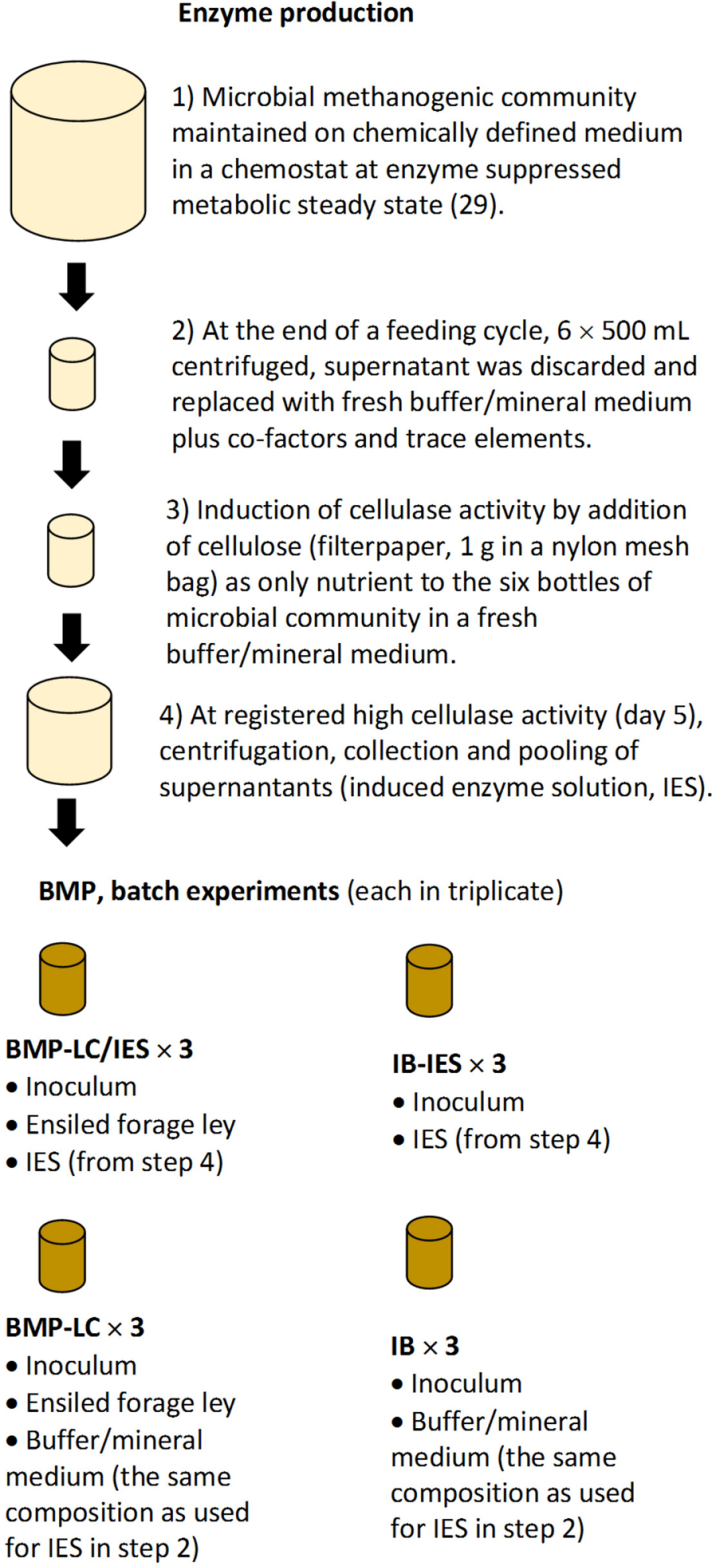
Flowchart over experimental procedure. For details please refer to “Methods”. For detailed information about the microbial methanogenic community at metabolic steady-state and induction of cellulases please see [29]. *IES* induced enzyme solution, *BMP* biochemical methane potential, *LC* lignocellulose, *IB* inoculum background

Earlier experiments of enzyme induction from the enzyme suppressed metabolic steady-state were performed directly in the continuously operated experimental biogas reactor [29]. However, in the present study, to avoid the transfer of any constitutively expressed enzyme or undigested organic material from the biogas reactor, which could potentially influence the results of a BMP test, the following procedure was followed (see Fig. 1 for an overall flowchart). At the end of a feeding cycle, 24 h from the last feeding with chemically defined medium in the bioreactor, samples of 6 × 500 mL culture were collected and transferred to N_2_-purged centrifugal bottles and centrifuged for 30 min at 9000×*g* and 37 °C. After centrifugation, the supernatant was discarded and each cell pellet was resuspended in 500 mL degassed and pre-heated (37 °C) buffer/mineral solution (i.e., medium without nutrients) before transfer to N_2_-flushed 1 L glass bottles. For induction of cellulases, 1 g of cut filter paper was added to each bottle in closed bags of nylon mesh as the sole carbon source (Whatman no. 1, Whatman Ltd, USA). The buffer/mineral solution had exactly the same composition as the chemically defined medium described earlier [29]. The pH was adjusted to 7.5 with 1 M NaOH, and the volume was adjusted with degassed Milli-Q water before being transferred to an airtight container. Prior to use, the pH was tested and adjusted if necessary. By this washing of cells and exchange of medium, no or very little organic material besides the added filter paper was present in the batch cellulase induction. To prevent degradation of any induced cellulases due to proteolysis by any endogenous proteases, one tablet of protease inhibitor cocktail was added to each bottle (cOmplete™, Roche Diagnostics, Mannheim, Germany). The bottles were sealed with a rubber stopper and aluminum cap and the head space was flushed with nitrogen. To the cell suspension, 500 µL of each vitamin, trace element and ultra-trace element stock solutions was added as described earlier [29]. The samples were incubated at 37 °C in a heated cabinet without agitation and the gas production, methane content, pH and cellulase activity were analyzed at defined time points. Cellulase activity was monitored using a fluorescent cellulase assay kit [30] (Marker Gene Technologies Inc. Eugene, USA) as earlier described [29]. Two days after cellulase activity was detected (day 5), the induction experiment was terminated. At the same time, a gas sample was collected from each batch bottle for analysis of the methane content. To prevent the transfer of any microorganisms to the BMP test, supernatants containing the induced extracellular cellulolytic enzymes were collected after 30 min centrifugation at 12,000×*g* and 37 °C of the cell suspension. This relative centrifugal force and time was considered sufficient to sediment even the smallest cellular microorganisms [31]. The cell-free supernatants, i.e., buffer/mineral solution containing induced cellulolytic enzymes, were pooled to a total volume of 2.95 L and then used as an induced enzyme solution (IES) in the following BMP test of lignocellulosic material.

### Biochemical methane potential tests of lignocellulosic material

The substrate, ensiled forage ley, and microbial inoculum were collected from a biogas production plant treating mainly energy crops (Swedish Biogas International, Örebro, Sweden). The ensiled substrate was collected directly from inside the covered silage windrows and preserved in a sealed plastic bag at 4 °C for 2 weeks prior to the experiment start. The inoculum was incubated for 1 week at 37 °C in an airtight container in which produced gas was vented via a water trap to allow as much residual substrate as possible to decompose before the experiment start. Prior to conducting the experiment, the total solids (TS) and volatile solids (VS) of the inoculum and the substrate were determined (Table 1). Determination of total solids (TS) and volatile solids (VS) for the ensiled forage ley and the inoculum was performed in accordance to Swedish Standard protocol SS 28113 [32]. The organic load of the ensiled forage ley was 3 g VS/L, and the VS ratio between the inoculum and substrate was set to 2:1. The active volume in each bottle was 300 mL (in 544 mL bottles) and the organic load from the inoculum was 1.8 g VS, and thus from the substrate 0.9 g VS (Table 1).

**Table 1:** TS/VS values and amount of used lignocellulosic substrate and inoculum in batch BMP experiments

	TS (% w/w)	VS (% of TS)	Amount added to each bottle (g)	Final amount VS (g)
Ensiled forage ley	19.7	88	5.2	0.9
Inoculum	6.9	77	33.9	1.8

All bottles were flushed with N_2_ gas prior to the start of the experiment to provide an oxygen free environment. For the BMP tests of the lignocellulosic substrate, there were four series in total for: BMP of lignocellulosic ensiled forage ley in buffer/mineral medium (BMP-LC); BMP of ensiled forage ley + induced enzyme solution (BMP-LC/IES); inoculum background production in buffer/mineral medium (IB), and inoculum background production in induced enzyme solution (IB-IES). For a full outline of the batch experiments, see Table 2 and Fig. 1. Each series was prepared separately and conducted in triplicate. The substrate and inoculum were added to the bottles along with 300 μL of vitamin, trace element and ultra-trace element stock solutions to the same final concentration as used in the experimental biogas reactor and induction experiment [29]. The volume was adjusted to 300 mL using either the buffer/mineral medium (for BMP-LC and IB) or the induced enzyme solution, already in the buffer/mineral medium of the same composition and pH (for BMP-LC/IES and IB/IES). The bottles were sealed with a rubber septum and the head space purged with N_2_. A 1 mL sample was collected from each bottle, the pressure was released from the bottles and the samples were incubated at 37 °C.

**Table 2:** Experimental setup for biochemical methane potential assay

Series	Inoculum	Ensiled forage ley	Buffer/mineral medium^a^	Induced enzyme solution^a^
BMP-LC × 3	●	●	●	
IB × 3	●		●	
BMP-LC/IES × 3	●	●		●
IB-IES × 3	●			●

^a^Each to a final volume of 300 mL in the respective setup

### Biomethane production

For the BMP test, the gas pressure during the initial high rate of gas production (first week) was determined daily. Later, when the gas production rate had decreased, gas pressure reading and sampling were performed after increasingly longer periods. The pressure was measured using a pressure gauge (Testo 312-3, Testo AG, Germany) before any other sampling. Before each pressure reading, the bottles were gently agitated (turned three times) to release any gas trapped in the particulate suspension. After sampling, the gas pressure was released and allowed to equalize to atmospheric pressure. The amount of produced gas and methane was calculated by considering the volume of the headspace in the bottles and converted to SI standard conditions. The methane concentration was determined by collecting 2.5 mL of gas from the batch bottle head space after pressure reading. The analysis was performed using gas chromatography (GC-FID, Clarus 500, Perkin-Elmer, Waltham, USA). 100 µL of sample was injected via a loop into a Porapak T80/100 mesh column (Perkin-Elmer, Waltham, USA) with N_2_ as carrier gas at 80 °C and a flow rate of 44 mL/min. All measurements were performed in duplicate.

### Data analysis

All fitting of the data and kinetic modeling were performed using a nonlinear least-squares program (TableCurve, Jandel Scientific, San Rafael, USA). The kinetics of biomethane production of both samples were fitted to the sum of two exponential first-order terms with the rate constant parameters in units of day^−1^ (Table 3; Fig. 4). The difference (decrease or increase) in biomethane production between the two samples (BML-LC/IES and BMP-LC) was fitted to single-phase, first-order exponential kinetics (Table 4; Fig. 6a).

**Table 3:** Amplitudes and rate constants for the two phases, first-order kinetics

Process	*A*_1_ (mL/g VS)	*k*_1_ (day^−1^)	*A*_2_ (mL/g VS)	*k*_2_ (day^−1^)	*A*_tot_	*R* ^2^
Overall BMP-LC/IES	239	0.242	86	0.044	326^a^	0.995
Overall BMP-LC	230	0.230	73	0.016	303^b^	0.991

Fitted to the equation $y = A_{1} \left( {1 - {\text{e}}^{{ - k_{1} x}} } \right) + A_{2} (1 - {\text{e}}^{{ - k_{2} x}} )$

^a^Experimentally determined final net production value at day 111 was 319 mL

^b^Experimentally determined final net production value at day 111 was 286 mL

**Table 4:** Amplitude, rate constant and half time for single-phase, first-order kinetics fitted to the decrease and increase in gas production

Process	Equation	*A* (mL/g VS)	*k* (day^−1^)	*t*_*½*_ (day)	*R* ^2^
1st phase decrease	$y = A \cdot {\text{e}}^{ - kx}$	51.4	0.432	1.6	0.986
2nd phase increase	$y = A \cdot \left( {1 - {\text{e}}^{ - kx} } \right)$	19.5	0.104	6.7	0.972

Half time (*t*_½_) was calculated for first-order kinetics using the relationship $t_{{{1 \mathord{\left/ {\vphantom {1 2}} \right. \kern-0pt} 2}}} = \frac{\ln 2}{k}$

## Results and discussion

### Induction and harvesting of cellulolytic enzymes

**Fig. 2:**
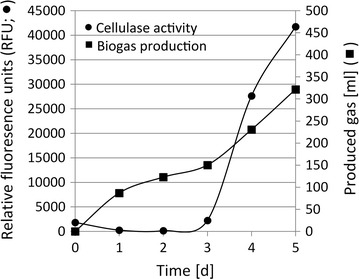
Time course of cellulase activity and biogas production of the microbial community collected from the steady-state bioreactor after washing, change of medium, transfer to batch bottles and cellulase induction with cellulose. The cellulase activity presented represents the final fluorescence reading after 120 min incubation with the fluorogenic substrate. Induction was terminated on day 5 to collect the induced enzyme solution (IES)

The BMP test of ensiled forage ley was used to analyze the effect that the induced endogenous cellulolytic enzymes had on the anaerobic digestion of a lignocellulosic material. For this purpose, a “clean” sample with low organic content but high enzymatic activity is desirable. However, in the steady-state reactor, which had an average hydraulic retention time of 31 days [29], the extracellular environment likely contained many components, such as proteins, cell debris, non-consumed substrate, metabolites, etc. Hence, owing to the organic content, it would not have provided an appropriate enzyme solution sample for the BMP test. Therefore, to still make use of the findings of targeted enzyme induction in the steady-state reactor, an aliquot of microorganism suspension from the steady-state reactor was collected by centrifugation and resuspended in pure buffer/mineral medium with no nutrients. This served several purposes. First, to create an environment deficient of 6-carbon sugars, which forces the microbial community to produce cellulases to make this nutrient available from the filter paper. A distinct increase in cellulase activity was registered after 3 days under the new culturing conditions in batch (Fig. 2), similar to earlier experiments conducted directly in the steady-state reactor [29]. Concomitantly, an increase in produced gas and a discoloration from yellow affinity compounds [29, 33] of the filter paper was registered. To confirm that the full methanogenic microbial community was still viable after the washing and medium change, the methane content in the head space was analyzed and the six samples from the batch inductions showed an average methane content of 23.8%. Since methane is the end product of complex syntrophic methanogenesis, it was concluded that all microorganisms necessary for the conversion of cellulose to methane were viable and active after washing and transfer to the batch culture. This was further confirmed by a registered stable pH during the cellulase induction, thus, indicating that there was no accumulation of fatty acids. After 5 days, i.e., 2 days after cellulase production had commenced, a very high cellulase activity was recorded and the cellulase induction experiment was terminated (Fig. 2). The nylon mesh bag with remaining filter paper was removed and the supernatant was collected by centrifugation and the cell pellet discarded. The remaining cell-free supernatant, with high extracellular cellulase activity in the buffer/mineral solution, was then used as an enzyme augment for the following BMP test with lignocellulosic material as substrate.

### Biogas production of samples and inoculum background

**Fig. 3:**
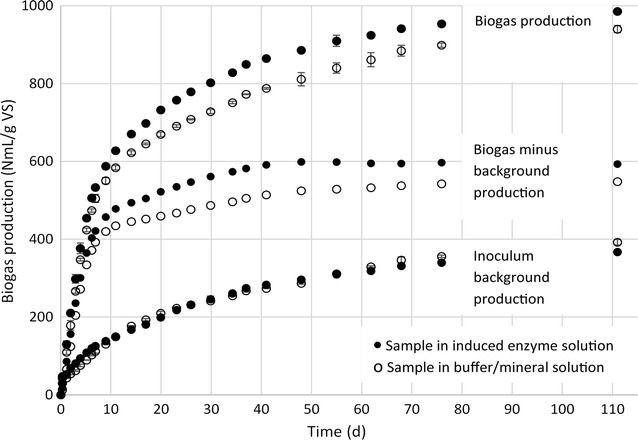
Raw data of accumulated biogas production in all BMP samples and the calculated net biogas production over the entire time period. *Error bars* represent actual ±1 standard deviation calculated from triplicate measurements

The second purpose of exchanging the medium of the steady-state reactor by a pure buffer/mineral medium for the induction of cellulases was to avoid subsequent transfer of large amounts of organic material with the supernatant to the BMP test. An observed increase in gas production from contaminating organic material could otherwise be misinterpreted as an enzymatic effect, especially if the biochemical methane potential of the substrate is low [25]. Evidently, by this approach, no or very little organic material besides the induced cellulases was transferred. In the biogas production raw data (Fig. 3), this was demonstrated by the fact that the two inoculum background references, one in only buffer/mineral medium (IB) and the other in buffer/mineral medium with induced enzymes (IB/IES), produced gas with no significant difference in amount. Thus, this control experiment confirmed that the medium with induced enzymes did not contain any significant amount of additional organic material compared to the sole buffer/mineral medium. Hence, the higher gas production rate and yield noted in the raw biogas production data of the BMP test of lignocellulosic material augmented with endogenous cellulases, as compared to the unaugmented process (Fig. 3), was not the result of simply adding more organic material to the enzyme augmented series (BMP-LC/IES). To compare the two BMP tests against each other, the non-influenced inoculum reference was used to subtract the inoculum background biogas production from BMP-LC and BMP-LC/IES (Fig. 3). Importantly, there was in neither case a lag phase in biogas production, which indicates that the 2:1 ratio between inoculum and substrate VS provided enough microorganisms, in the correct composition, not to cause a rate limiting step *en route* to production of biomethane.

### BMP results

#### Biomethane concentration

Methane concentration was determined intermittently once a week. Since the biogas production rates were high, already by the first methane measurement (day 7) approx. 533 and 504 mL of biogas had been produced for BMP-LC/IES and BMP-LC, respectively (Fig. 3), and the methane concentrations had reached values of 46.7 ± 0.1% and 43.7 ± 0%, respectively. By the second methane concentration sampling time point (day 14), the methane concentration had plateaued for both samples, as judged by the following measurements. To determine the average methane concentration, this and the following eight methane concentration values were used, giving mean concentrations of 51.9% (with a standard deviation of 2.4) and 49.4% (SD = 1.4) for BMP-LC/IES and BMP-LC, respectively. These results suggest a slightly higher methane concentration in the sample with induced enzyme solution. However, as indicated by the standard deviation between the measured time points, it was not possible to assign a significantly higher methane concentration to the IES supplemented sample. Therefore, to recalculate the biogas production to biomethane production, final methane concentrations values determined at day 111 were used (53.8 ± 0.1% and 52.2 ± 0.1% for BMP-LC/IES and BMP-LC, respectively). These values are in accordance with the theoretical concentration of 50% methane in biogas produced from polysaccharides [34], thus indicating that the vast majority of biomethane was produced from cellulose and hemicellulose in the substrate. The small difference in methane concentration is also consistent with the conclusion that insignificant additional organic materials, in the form of proteins and lipids, were supplied by the induced enzyme solution. These substrates otherwise produce biogas with a significantly higher theoretical biomethane concentration in the gas composition of approx. 60 and 70%, respectively [34]. Using the final methane concentration at day 111, values for the actual final BMP of the accumulated biomethane were determined, i.e., 319 and 286 NmL CH_4_/g VS for BMP-LC/IES and BMP-LC, respectively, corresponding to a difference of +12% at day 111 in the sample supplemented with the induced enzyme solution. It is, however, difficult to relate the results to those from other BMP tests, because in most other studies, the “BMP” values presented are not the actual final biochemical methane potential reached after biomethane production has ceased. Often, BMP tests are terminated after 25–35 days, although it is obvious from experimental results that in most cases, this is not enough to allow complete digestion of lignocellulosic material [35]. Furthermore, in industrial plants, the residing time is most often considerably longer [4]. Therefore, unless the value is collected after the net biomethane production has ceased, BMP values should be more correctly presented as BMP_*x*_, where *x* denotes the number of days incubated. Nevertheless, the BMP_111_ of 286 NmL CH_4_/g VS for the untreated ley forage silage is a reasonable value. This value is slightly lower than BMP_35_ of untreated whole crop rye and maize silage, which ranges between 305 and 341 NmL CH_4_/g VS [21, 23]. This lower value of BMP-LC for untreated ley forage silage is to be expected because the BMP value of whole crop silage includes the BMP of the energy rich kernel of energy crops. However, compared to more similar substrates, the value of 286 NmL CH_4_/g VS for BMP-LC is generally higher. Such substrates include, e.g., harvested switch grass [9], miscanthus [11], ensiled sorghum forage [12] and Kanlow switch grass [14], for which BMP_30_ is reportedly 205, 136, 265, and 197 NmL CH_4_/g VS, respectively. Part of this difference can be explained by the early termination of these experiments after 30 days, when the final full biochemical methane potential had not yet been reached. Regarding the final BMP, the value for untreated ley forage silage was closest to that of completely digested wheat straw, which shows BMP ranging between 233 and 316 NmL CH_4_/g VS depending on the inoculum used [35].

**Fig. 4:**
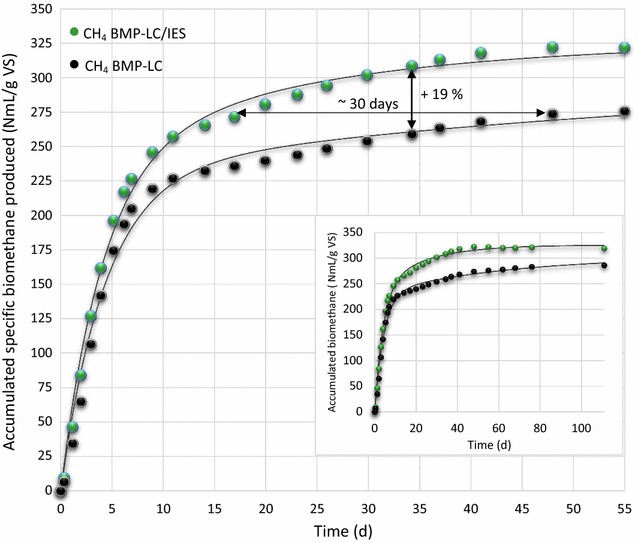
Accumulated specific biomethane produced from ensiled forage ley in the enzyme augmented sample and the untreated reference after subtraction for background production from the untreated inoculum reference. *Solid lines* represent fitted data using the equation and values in Table 3

Therefore, overall, the absolute calculated value of BMP was reasonable. The final methane concentration was used to recalculate biogas production to biomethane production (Fig. 4). However, to indirectly calculate the kinetics of biomethane production over the entire time course from the final methane concentration, one needs to assume that the same substrate composition is being degraded over the whole time range. This was supported by the consistently low methane concentration of approx. 50% derived from almost exclusively anaerobic digestion of polysaccharides. Therefore, the assumption that biomethane was produced from the same substrate composition over the entire BMP test period was judged to be reasonable.

#### Biomethane production rate and yield

For both samples, the biomethane production could be divided into two phases (Table 3). The first fast phase (from the start to approx. day 9) represented gas production from the hydrolysis of easily accessible material. Notably, kinetic analysis of the biogas production rate (Table 3) revealed that the first phase of the enzyme augmented biomethane production was only slightly faster and the amplitude of the first fast phase was almost identical for the enzyme augmented and the untreated sample. This further indicates that no additional and easily accessible organic material was transferred with the induced enzyme solution. Since the amplitude and rate constant of the first fast phase were almost identical between the two samples, this phase most likely represents the digestion of material that is easily accessible and digested to the same degree irrespective of whether added enzyme is used or not. The slower second phase is more relevant in terms of the effect from adding endogenous enzymes as it represents the biomethane production from digestion of less microbially accessible material, such as lignocellulose. It was further evident that the biomethane production rate in the second phase of the experiment augmented with the induced enzyme solution added (BMP-LC/IES) was significantly faster than without enzyme (BMP-LC) with a rate constant almost three times as high in the second slower phase (Table 3).

Although the differences in rate constants were relatively low, these differences still had a strong effect on biogas production owing to the timescale of anaerobic digestion of lignocellulosic material. This was especially noticeable from approx. day 7, when the first phase has started to decline and the process became dominated by the second phase from the digestion of less accessible material and the biomethane production in the two cases started to diverge (Fig. 4). Thus, by day 34, when the process supplemented with enzyme had consumed almost all (96%) of the substrate, the accumulated biogas was 19% higher than in the process without added enzyme (Fig. 4). Notably, by day 48, all substrate in the enzyme augmented sample had been consumed and at subsequent time points, no net biomethane production in the enzyme augmented BMP-LC/IES sample was observed (see inset in Fig. 4). However, the sample without added endogenous enzymes (BMP-LC) continued to produce gas because the available fraction of substrate, which was exactly the same as in the enzyme augmented sample, was not yet fully consumed owing to the lower hydrolysis rate. Thus, at later time points, the net biomethane production between the two samples started to converge and the final difference at BMP_111_ was only 12%. It should be noted that this result would not have been registered if the experiment had been terminated after 35 days. However, this result is as expected from the addition of enzymes, with no extra organic substances added, because enzymes merely increase the rate of reaching equilibrium.

**Fig. 5:**
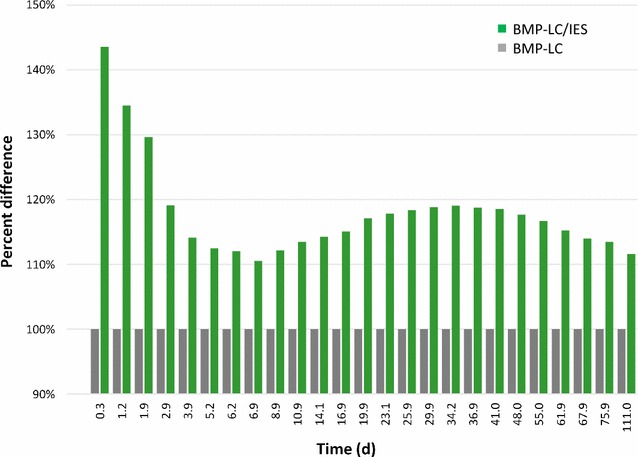
Percent difference in gas production between the enzyme augmented sample and the untreated sample normalized to 100%. Note that the *x*-axis is not linear but represents actual time points for gas production sampling (more frequent in the beginning)

**Fig. 6:**
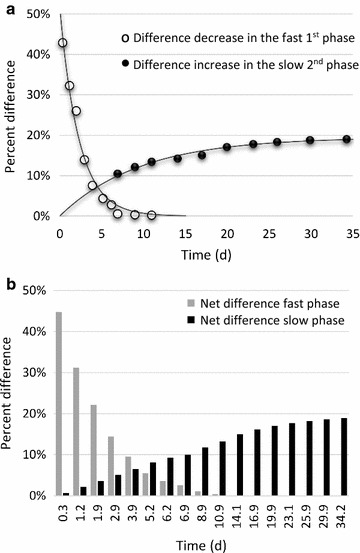
Difference decrease in the fast 1st phase and difference increase in biogas produced from the slow 2nd phase. **a** Kinetic analysis and fitted data (*solid line*). Analysis was for the slow 2nd phase based on values recorded between day 7 and day 34 in Fig. 5. **b** Resulting percent net differences at different time points for the separated initial fast phase (*grey bars*), and the second slow phase (*black bars*) of BMP-LC/IES as compared to BMP-LC. At approximate day 9, there is no net difference in biogas production from easily accessible material consumed in the fast phase. In contrast, in the slow phase of biogas production from hydrolysis of less accessible material, the net difference between BMP-LC/IES and BMP-LC increases until day 34. To facilitate comparison to the non-separated experimentally collected data, **b** is plotted with the same time evolution as in Fig. 5

The above result was even more discernible when the percent difference in gas production between the two samples was plotted (Fig. 5). There was a noticeable difference in gas production rate in the first phase, during which easily accessible material in the substrate was rapidly hydrolyzed by the enzymes in BMP-LC/IES, and subsequently consumed. However, since this part of the substrate was hydrolyzed and consumed only slightly slower in the sample without endogenous enzymes added, this difference in biomethane production declined rapidly with time. It should be noted that this difference in the initial phase was based on low absolute values (Fig. 4). By approx. day 7, the percent difference in net biomethane production again started to increase, and this trend continued until essentially all substrate in the enzyme augmented sample had been consumed. Therefore, at later time points (from day 34, Fig. 5), the difference in gas production again started to decline due to the ceased net gas production in the enzyme augmented sample and the continued net gas production in the sample without added enzyme. Thus, the data show that two processes operated simultaneously on two different fractions of the substrate. Of these, the first is less interesting because it is the process of degrading easily accessible material. Thus, although the enzymes started hydrolyzing also the more recalcitrant part of the substrate already from day 1, this process was obscured by the simultaneous high gas production from the more easily accessible material. To determine the actual net effect on gas production of the induced and added endogenous enzymes on the hydrolysis of the recalcitrant lignocellulosic material, the kinetics of the difference in gas production was calculated using only data for the slow phase between day 7 and day 34 (Fig. 6a). This yielded an almost perfect fit to a one phase first-order kinetic process with an amplitude of 19.5%, a first-order rate constant of 0.104 day^−1^ and a half time (*t*_½_) of 6.7 days (Table 4). By subtracting the small calculated value of the fitted data for the slow second phase from each time point in the first phase (between day 0 and 7), the kinetics for degradation of the easily digested material in the first phase could also be estimated (Fig. 6a; Table 4) and the two simultaneous processes separated (Fig. 6b).

These results suggest that for treatment of the easily accessible fraction of the material, enzyme augmentation is unnecessary if the residence time is more than approx. 9 days because after this time, the net difference between the two samples was minimal (Fig. 6a, b). However, the induced enzyme solution aided in the digestion of less accessible material up to almost completely consumed ensilaged forage ley (Fig. 6b) over a time period of approx. 34 days. This is perhaps the most striking result in this study because it imply that the enzymes in the induced enzyme solution, added only at the start of the BMP test, were active throughout the BMP test. This contrasts with earlier studies using addition of commercially available enzymes to the anaerobic digestion environment of sludge treatment, in which it was shown that no enzyme was active or influenced the biogas production rate for more than approximately 24 h [25]. This is important because enzymes with high longevity in the environment of anaerobic digestion would allow for less and fewer additions of fresh additions of enzyme.

In summary, addition of the induced enzyme solution led to an increased rate of biomethane production and higher yield of biomethane, which culminated on day 34 (Figs. 5, 6) with a maximum of 19% higher biomethane production in the enzyme augmented sample. In practical applications, this enzymatically increased biogas production rate opens up possibilities for two different strategies. First, the main goal could be to extract the maximal amount of biomethane per unit mass of substrate. For the enzyme augmented process, this occurred at approx. day 48, when there was no longer net biomethane production in the enzyme augmented sample (inset Fig. 4). At the same time, the BMP of the untreated process only reached 85% of that of the enzyme treated sample. Alternatively, the main goal could be to produce as much biomethane as possible per unit reactor volume. For example, it could be that 85% utilization of the substrate is considered to be enough. For the untreated sample, this occurred at approx. day 48, whereas for the enzyme treated process this was achieved by approx. 15–20 days, i.e., approx. 30 days faster (Fig. 4). Thus, in theory, it may be possible to achieve doubled turnover and production of biogas, at 85% yield, with the use of the induced endogenous enzymes.

## Conclusions

The enzymes in the induced enzyme solution (IES) predominantly enhanced the rate of biomethane production, suggesting that the enzymes increased the degradation rate of lignocellulosic biogas substrate. The results also showed that it is, thus, possible to influence the biogas production rate from lignocellulosic substrates by addition of appropriate enzymes directly to an anaerobic digester. This would eliminate the need for auxiliary energy intensive processes, thereby potentially avoiding other high capital or operating expenses. Most strikingly, the effect of the induced enzyme solution was maintained over the whole time period of the process, indicating that the induced enzymes were stable and active in the anaerobic digestion process over the same time span. Such enzymes could of course be of large value for the in situ treatment of lignocellulosic substrates to increase biogas production rates and yields provided they can be identified, cloned, and produced at low cost in recombinant systems [8]. However, owing to the limitations of the cellulase activity assay, it was not possible to unambiguously assign the effect to endo- or exo-cellulases, β-glucosidases or other auxiliary synergistic proteins [36]. Therefore, to enable identification of the responsible proteins, a thorough analysis of the extracellular induced enzyme solution need to be performed.

Such analysis is complicated by the fact that the enzymes are produced by member(s) of a mixed microbial community of unknown structure. However, because the methanogenic microbial community was maintained on a chemically defined medium under controlled conditions, a metaproteomic analysis of the extracellular proteins was made possible [37]. Recently, using the described approach to preferentially and distinctly induce cellulolytic enzyme activity [29] the resulting clear differences in the protein expression pattern between the induced and the non-induced state were used to pinpoint a number of proteins that were upregulated in response to the need to hydrolyze cellulose [38]. As expected, by this study it was found that the induced enzyme solution contained several enzymatic functionalities related to the degradation of cellulose and hemicellulose. These included several actively secreted cellulases, xylanases, and β-glucosidases as well as cell wall anchored cellobiose phosphorylases. In addition, several hydrogen peroxide producing enzymes (copper amine oxidases) were strongly upregulated, implying that members of the microbial community produced enzymes possibly involved in lignin degradation. The majority of the enzymes could be assigned to be produced by species closely related to, but not unambiguously identical with, *Ruminiclostridium thermocellum* and *Clostridium straminisolvens*. However, because of the lack of identical enzyme counterparts in public databases, and the more severe protein inference issue of metaproteomics, no exact identification of the complete amino acid sequence could be accomplished for any of the enzymes. Thus, in order to clone, produce and characterize the correct variants of these prospective novel enzymes further studies are needed [38].

## Abbreviations

BMP: biochemical methane potential
IES: induced enzyme solution
TS: total solids
VS: volatile solids
LC: lignocellulose
IB: inoculum background
SI: Système International
GC-FID: gas chromatography-flame ionization detector
NmL: mL gas normalized to standard SI conditions
